# Clinical characteristics of cystic encephalomalacia in children

**DOI:** 10.3389/fped.2024.1280489

**Published:** 2024-05-22

**Authors:** Lijuan Fan, Lianying Feng, Jing Gan, Rong Luo, Haibo Qu, Xiaolu Chen

**Affiliations:** ^1^ Department of Pediatrics, West China Second University Hospital, Sichuan University, Chengdu, Sichuan, China; ^2^ Key Laboratory of Birth Defects and Related Diseases of Women and Children, Sichuan University, Chengdu, Sichuan, China; ^3^ Key Laboratory of Development and Maternal and Child Diseases of Sichuan Province, Sichuan University, Chengdu, Sichuan, China; ^4^ Department of Neurology, Children’s Hospital of Chongqing Medical University, Chongqing, China; ^5^ Department of Radiology, West China Second University Hospital, Sichuan University, Chengdu, Sichuan, China

**Keywords:** cystic encephalomalacia, clinical manifestations, medical imaging, MRI, pediatrics

## Abstract

**Purpose:**

To investigate the primary causes and clinical characteristics of cystic encephalomalacia (CE) in children.

**Methods:**

The clinical data of 50 children who were admitted to our hospital due to CE between January 2008 and December 2020 were retrospectively reviewed. Their primary causes, clinical manifestations and cranial magnetic resonance imaging features were analyzed.

**Results:**

Among all patients, 5 had prematurity, 19 had hypoxic-ischemic encephalopathy (HIE), 13 had intracranial infection, 14 had traumatic brain injury and hemorrhage, 4 had cerebral infarction, 2 had congenital genetic diseases, and 1 had hypoglycemia. The average time from primary disease onset to CE diagnosis was 70.1 ± 61.0 days. The clinical manifestations included speech or motor developmental delay (*n* = 33), epilepsy (*n* = 31), dystonia (*n* = 27), limb paralysis (*n* = 16), and visual or auditory impairment (*n* = 5). Patients with HIE as the primary cause of CE had a significantly higher occurrence of dystonia, while a significantly higher incidence of paralysis was observed in those with cerebral infarction as the primary cause.

**Conclusion:**

CE in children is mainly caused by HIE, intracranial infection, and cerebral hemorrhage. The major clinical manifestations included speech or motor developmental delay, epilepsy, and dystonia. Magnetic resonance imaging is an important tool for the diagnosis of CE.

## Introduction

Cystic encephalomalacia (CE), referring to cystic degenerative spaces of the cerebral matter after injury, results in decreased consistency of brain tissue. It is associated with a focal increase in the amount of cerebrospinal fluid, the residuals of liquefactive necrosis, and hemosiderin (1, 2). CE poses a serious threat to the health of patients, it leads to necrosis and cystic degeneration of brain tissue, which may cause various serious neurological symptoms and complications. It may cause movement disorders, sensory abnormalities, increase the risk of epileptic seizures, affect cognitive function, lead to intellectual decline, memory decline, and seriously affect the patient's learning ability and social function. Despite some identified etiology, our understanding of the risk factors of cerebral cystic necrosis remains limited. Fetuses and neonates are more prone to develop CE, even multicystic encephalomalacia, due to high amounts of premyelinating oligodendrocytes, N-methyl-D-aspartate receptors, and water content in the immature brain (3–5). A child's brain was considered more plastic than that of an adult, and early brain damage tended to have better outcomes (6). However, recent studies have revealed that the younger the age of onset, the worse the prognosis, which places a huge burden on society and families (7–9). Moreover, children with CE often develop neurological deficits years after the disease onset (10). Patients may face problems such as decreased self-care ability, limited social skills, and emotional disorders in the later stage, resulting in a significant decline in their quality of life. The recovery of neurological function after CE is closely related to the location of injuries (11). Previous studies have reported the correlations between the site of brain injury and specific manifestations, such as cerebral palsy, epilepsy, developmental delay, and intellectual disability (8, 12–16). But the varied clinical presentations of CE, coupled with overlapping features with other neurological disorders, often hinder precise diagnosis. The absence of specific biomarkers and imaging modalities further exacerbates diagnostic challenges. If some specific clinical manifestations could be elucidated or certain risk factors leading to CE identified, it would greatly aid in the early detection of CE and improve prognosis.

Magnetic resonance imaging (MRI) is a non-invasive, radiation-free imaging technique widely used in the diagnosis of severe brain injury owing to its high sensitivity and specificity for detecting CE of different causes (17). However, there is no consensus on the timing of the MRI in predicting prognosis of CE. For neonates with hypoxic-ischemic encephalopathy (HIE), the Guidelines of the American Academy of Neurology recommend MRI on postnatal days 2–8, while the Guidelines of the American College of Obstetricians and Gynecologists state that MRI should be performed in both early (1–4 days) and late (7–21 days) postnatal periods to obtain a more comprehensive assessment of injuries (15–18). As CE is a dynamic process and a child's brain undergoes growth and development, it holds significant importance to conduct MRI scans during follow-up for children with severe brain injuries. This enables better tracking of CE characteristics and its correlation with clinical manifestations, such as language, audiovisual perception, cognition, muscle tone, and the presence of combined epilepsy.

In this study, we conducted a retrospective analysis to assess the primary etiologies, clinical manifestations, and cranial MRI characteristics of childhood CE. Our objective was to delineate the prevalent causes of CE, while also examining the association between the primary causes, presenting symptoms, and location of brain lesions identified in MRI scans. The overarching aim is to enhance early diagnosis, prognostic assessment, and management strategies for CE.

## Materials and methods

### Patients

Children who were diagnosed with CE according to the criteria proposed by Schmitt (1) and confirmed by the MRI examination between January 2008 and December 2020 at the West China Second Hospital, Sichuan University were screened for enrollment. The inclusion criteria were as follows: (1) Cranial MRI showed extensive T2 hyperintensity and T1 hypointensity, low or slightly high T2/FLAIR signal, no significant diffusion-weighted imaging restriction, no significant enhancement, and white matter gliosis around the cyst. Some cases may also show thinning corpus callosum or other brain atrophy-related changes; (2) The time from the onset to the last follow-up was over 6 months and the medical history was complete. Patients who underwent surgery or radiotherapy, had parasites (e.g., Neurocysticercosis, Toxoplasmosis, Hydatid disease) were excluded. Finally, 50 children with CE were recruited. Ethical approval for this study was obtained from the institutional review board of the Public Health and Clinical Center of Chengdu (West China Second University Hospital of Sichuan University).

### Data collection

The medical history, results of laboratory and genetic tests, the cell count, the pathogen analysis of cerebrospinal fluid, the electroencephalogram data, and the results of cranial MRI of all subjects were collected. The onset of neurological symptoms, such as altered consciousness, seizures, and paralysis, is typically defined as the time when they first manifest, either based on family reports or medical observation. The moment of diagnosis is determined by the first detection of CE changes on MRI scans. The cranial MRI results were individually reviewed by two experienced radiologists. The brain was divided into the right and left cerebral hemispheres, which then further divided into six regions, including the frontal lobe, parietal lobe, temporal lobe, occipital lobe, basal ganglia and thalamus, and cerebellum or brainstem. The location and degree of the encephalomalacia foci were assessed. The clinical manifestations of CE, including epilepsy, limb paralysis, dystonia, speech or motor developmental delay, and audiovisual impairment, were recorded by follow-up visits or telephone follow-ups.

### Statistical analysis

Data were analyzed using the SPSS software version 28.0 (IBM, Armonk, New York, USA). Categorical data were shown as frequency (%) and compared by Fisher's exact test. Data that followed a normal distribution or an approximately normal distribution were expressed as mean ± standard deviation compared by ANOVA. A *P*-value of less than 0.05 was considered statistically significant.

## Results

### Basic information

There were 31 males and 19 females, with a male-to-female ratio of 1.63:1. Among all patients, 28 were aged 0–28 days, 20 were aged 29 days–3 years, and 2 were aged over 3 years when the manifestation first onset. The median age of onset was 47.5 days. The mean time from onset to the diagnosis of CE was 70.1 ± 61.0 days. The average follow-up time was 4 years and 3 months, with the shortest of 1 year and 1 month and the longest of 8 years and 4 months.

### Primary causes of Ce

According to the clinical data, the possible single or associated primary causes of CE included HIE (*n* = 19, 38%), traumatic brain injury/hemorrhage (*n* = 14, 28%), intracranial infection (*n* = 13, 26%), premature birth (*n* = 5, 10%), cerebral infarction (*n* = 4, 8%), genetic disorders (*n* = 2, 4%, both of them were incontinentia pigmenti), and neonatal hypoglycemia (*n* = 1, 2%). Seven children (14%) had unknown etiology.

**Figure 1:**
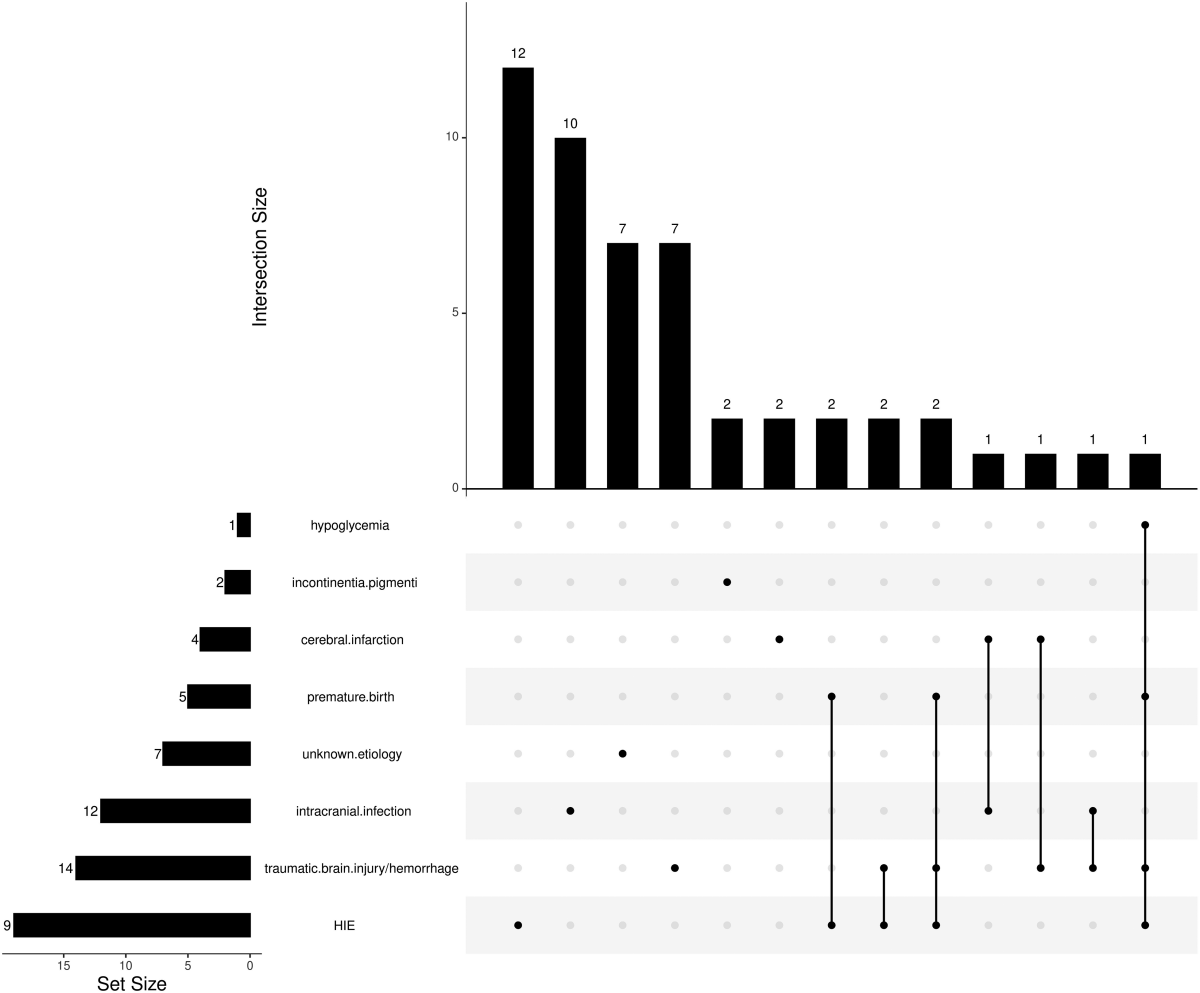
The UpSet plot of the primary causes of CE.

HIE was the primary cause in 60.7% of the patients with the disease onset of 0–28 days. Intracranial infection was the primary cause in 50% of the patients with the disease onset of 29 days–3 years, followed by intracranial trauma/hemorrhage (30%). There were 3 patients with the presence of at least three primary causes, 7 with two primary causes, and 33 with a known single primary cause. The intersections of the primary causes are shown in Figure 1. All preterm children had combined primary causes of CE, such as hypoxia-ischemia and cerebral hemorrhage.

Among the 13 children with intracranial infection, the pathogen of three (23%) was identified, including one *Streptococcus pneumonia*, one *Herpes simplex virus type 1*, and one *Enterovirus 71*.

### Clinical manifestations

The clinical manifestations of all children are shown in Table 1. There were 33 cases with speech or motor developmental delay, 31 with epilepsy, 27 with dystonia, 16 cases with unilateral or bilateral limb paralysis, and 5 with visual or auditory impairment. No significant difference in the time from symptom onset to diagnosis of CE was observed among groups with different primary causes (Table 2). A significantly higher occurrence of visual or auditory impairment was observed in preterm infants (*n* = 3, 66.0%) vs. term infants (*n* = 2, 4.4%) (*P *= 0.002), including strabismus, refractive error, deafness and abnormal auditory brainstem responses. The analysis of the clinical manifestations of 33 cases with a known single primary cause of CE showed that patients with HIE had a significantly higher occurrence of dystonia, while a significantly higher incidence of paralysis was observed in those with cerebral infarction (Table 3).

**Table 1:** Clinical manifestaions of CE in 50 children.

Clinical manifestations	Number	Frequency (%)
Speech or motor developmental delay	33	66%
Epilepsy	31	62%
Dystonia	27	54%
Limb paralysis	16	32%
Audiovisual impairment	5	10%

**Table 2:** Time from onset to the diagnosis in 33 cases with single primary cause of CE.

Primary causes	*N*	Time from onset to diagnosis (days, mean ± standard deviation)	*F*-value	*P*-value^a^
Hypoxic-ischemic encephalopathy	12	92.80 ± 71.605	0.780	0.549
Intracranial infection	10	53.38 ± 34.238		
Traumatic brain injury/hemorrhage	7	89.57 ± 95.591		
Cerebral infarction	2	25.50 ± 14.849		
Genetic diseases	2	48.00 ± 59.397		
Total	33	73.41 ± 67.508		

^a^ Statistical analysis was conducted using ANOVA.

**Table 3:** The number of CE patients with different clinical manifestations in each primary cause category^a^.

Primary causes		Epilepsy		*P* ^b^	Limb paralysis		*P*	Dystonia		*P*	Speech or motor developmental delay		*P*	Audiovisual impairment		*P*
		Yes	No		Yes	No		Yes	No		Yes	No		Yes	No	
Hypoxic-ischemic encephalopathy	Yes	5	7	0.273	2	10	0.249	10	2	0.010	8	4	1.000	1	11	1.000
	No	14	7		9	12		7	14		13	18		2	19	
Intracranial infection	Yes	7	3	0.455	3	7	1.000	8	2	0.057	7	3	0.710	2	8	0.212
	No	12	11		8	15		9	14		14	19		1	22	
Traumatic brain injury/hemorrhage	Yes	5	2	0.670	4	3	0.186	0	7	0.003^c^	4	3	0.686	0	7	1.000
	No	14	12		7	19		17	19		17	9		3	23	
Cerebral infarction	Yes	0	2	0.172	2	0	0.040	1	1	1.000	0	2	0.125	0	2	1.000
	No	19	12		5	26		16	15		21	10		3	28	
Genetic diseases	Yes	2	0	0.496	0	2	0.542	1	1	1.000	2	0	0.523	0	2	1.000
	No	17	14		11	20		16	15		19	12		3	28	

^a^ Only cases with singe primary cause were counted. The cause was inferred from clinical manifestations and examination results.

^b^ Fisher's exact test was used for statistical comparison.

^c^ The group with traumatic brain injury/hemorrhage as the primary cause of CE had significantly fewer patients with dystonia than other primary groups.

### EEG results

A total of 36 children underwent the EEG examination. Abnormal background activity was observed in 20 cases (55.6%) and the spike-wave was present in 29 cases (80.6%).

### Imaging assessment

The cranial MRI of all 50 children suggested CE, with unilateral lesions in 17 (34%) cases and bilateral lesions in 33 (66%) cases. The lesions were at all brain regions in 15 cases, at the parieto-occipital frontotemporal lobe in 4 cases, at the frontoparietal temporal in 6 cases, at the frontotemporal occipital in 1 case, at the frontoparietal occipital lobe in 5 cases, at the temporoparietal occipital lobe in 5 cases, at the frontoparietal temporal lobe in 5 cases, at the temporal lobe in 1 case, and at the frontal lobe in 1 case. There were 32 patients with lesions in the basal ganglia and thalamus and 4 patients in the cerebellar or brainstem.

The location of the cystic lesions in 33 cases with known single primary causes of CE was then analyzed and no significant difference in the number of patients with different lesion locations was observed among different primary cause categories (Table 4). Thirteen children underwent cranial Magnetic Resonance Angiography (MRA) examination and abnormalities were found in 12 cases, including 1 case with lesions at the proximal end of the intracranial artery, 4 cases in the anterior cerebral artery, 10 cases in the middle artery, and 4 cases in the posterior artery.

**Table 4:** The number of CE patients with different primary cause in each lesion site category^a^.

Lesion site		Hypoxic-ischemic encephalopathy		*P* ^b^	Cerebral infarction		*P*	Traumatic brain injury/hemorrhage		*P*	Cerebral infarction		*P*	Congenital genetic diseases		*P*
		Yes	No		Yes	No		Yes	No		Yes	No		Yes	No	
Basal ganglia & thalamus	Yes	8	15	0.327	7	16	1.000	5	18	1.000	1	22	0.521	2	21	1.000
	No	4	6		3	7		2	8		1	9		0	10	
Parietal lobe	Yes	12	17	0.271	8	21	0.567	7	22	0.555	1	28	0.231	1	28	0.231
	No	0	4		2	2		0	4		1	3		1	3	
Occipital lobe	Yes	9	13	0.703	7	15	1.000	4	18	0.661	0	22	0.104	2	20	0.542
	No	3	8		3	8		3	8		2	9		0	11	
Frontal lobe	Yes	11	16	0.379	7	20	1.000	6	21	1.000	1	26	0.335	2	25	1.000
	No	1	5		3	3		1	5		1	5		0	6	
Temporal lobe	Yes	12	18	0.284	8	22	0.212	6	24	0.523	2	28	1.000	2	28	1.000
	No	0	3		2	1		1	2		0	3		0	3	
Cerebellum or brainstem	Yes	0	2	0.523	1	1	0.521	0	2	1.000	0	2	1.000	1	1	0.119
	No	12	19		9	22		7	24		2	29		1	30	

^a^ Only cases with singe primary cause were counted. The cause was inferred from clinical manifestations and examination results.

^b^ Fisher's exact test was used for statistical comparison.

## Discussion

The male-to-female ratio of CE in this study was 1.63:1. Numerous studies have indicated that females exhibit a more favorable prognosis following severe brain injury, attributed to interactions between the immune and endocrine systems (19). This trend also extends to children with CE. Infancy emerges as the most susceptible period for CE, potentially due to an underdeveloped blood-brain barrier and immune system, as well as a higher incidence of accidental injuries. The primary causes of CE in children are diverse, including prematurity, HIE, hypoglycemia, craniocerebral trauma/hemorrhage, cerebral infarction, intracranial infection, and genetic disorders (20, 21). In this cohort, the most prevalent primary cause was HIE (38%), followed by traumatic brain injury and hemorrhage (28%) and intracranial infection (26%). The primary underlying diseases leading to CE in patients of different age groups are consistent with the most common neurological disorders prevalent in that age group.

The formation of cysts initiates with liquefactive necrosis in the brain parenchyma, lasting approximately 8–24 h, succeeded by an inflammatory phase lasting 3–5 days. This phase is characterized by macrophage infiltration, reactive astrocyte proliferation, and the development of axonal spheroids. Subsequently, the final phase, termed the chronic stage of recovery, ensues as a continuation of the inflammatory phase, marked by the formation of cystic cavities. This stage often presents with thinning of the corpus callosum, ventriculomegaly, and cerebral atrophy (22). In our patient cohort, the mean duration from symptom onset to diagnosis was 70.1 ± 61.0 days, with no significant variance noted among groups with distinct primary etiologies. Furthermore, there was no discernible correlation between the localization of cystic lesions and the identified primary cause. This lack of association may be elucidated by the overlapping effects of various primary pathologies on neuronal cell apoptosis and necrosis, such as compromised cellular energy metabolism, accumulation of metabolic byproducts and toxins, heightened generation of reactive oxygen species, and release of inflammatory cytokines and excitatory neurotransmitters (22). In our investigation, the shortest duration from symptom onset to diagnosis was 6 days. This case involves a newborn infant exhibiting characteristic blistering rash and experiencing frequent seizures shortly after birth, leading to a diagnosis of incontinentia pigmenti. A pathogenic mutation was identified in the *NEMO* gene subsequently. This instance suggests the potential utility of cranial MRI in early-stage detection of CE.

The incidence of neonatal HIE is 1–8 per 1,000 live births worldwide (23). Although the survival rates of preterm neonates and asphyxiated newborns have been increasing in recent years, 25% of them may have permanent neurological impairments, with cerebral palsy being the most common sequela, followed by developmental delay, cognitive impairment, epilepsy, and limb paralysis (23, 24). In our study, there were 14 full-term children with HIE, 10 of whom had abnormal muscle tone. Compared to patients with other primary causes, full-term HIE was significantly associated with abnormal muscle tone, which may be related to extensive involvement of the corticospinal tract (25). There are two major changes on the MRI of full-term children with HIE: (1) Patients with mild to moderate hypoxic ischemia show damages in the subcortical white matter and parasagittal watershed; (2) Patients with severe hypoxic ischemia exhibit damages in the basal ganglia, hippocampus, and sensorimotor cortex (26). In the present study, hypoxia-ischemia resulted in extensive subcortical encephalomalacia and basal ganglia lesions in all lobes, suggesting severe HIE. In the cohort of children with hypoxic-ischemic encephalopathy (HIE) exhibiting residual movement disorders, epilepsy, and developmental delay, only a subset are diagnosed with CE. Notably, there are significant discrepancies in manifestations, recovery duration, and long-term prognosis compared to patients without CE (27). Our study did not pursue comparative analyses in this domain, indicating a potential avenue for future research. Compared to full-term infants, those who are born preterm are at a higher risk of abnormal visual or auditory function, probably because the visual and auditory system is not well developed in premature infants, and they are predisposed to have jaundice, hypoxia and acidosis, which can cause secondary audiovisual damage (28, 29).

Intracranial infection induces brain damage mainly through local leukocyte infiltration, cytotoxic and vasogenic edema, neuronal necrosis, vasculitis, cerebral infarction, cerebral hemorrhage, increased intracranial pressure and herniation (30). Most patients with intracranial infection show no changes on MRI (31). In our study, there were 13 children with intracranial infection. One had *Streptococcus pneumonia* with clinical manifestations of convulsions, hemiparesis, and sensorineural deafness. The MRI suggested necrosis, cystic changes, and subdural effusion in the frontal, parietal and occipital cortex of the left cerebral hemisphere, possibly associated with multiple small vessel vasculitis. One had *herpes simplex virus type 1*, with temporal, parietal, and occipital cerebral softening during follow-up. One had *Enterovirus 71* infection, which caused severe hand, foot, and mouth disease. The cranial MRI showed diffuse cerebral softening and cerebellar atrophy throughout the brain, which was consistent with the previously reported imaging features (32).

The main manifestations of cerebral infarction in children include hemiparesis (50%–80%), speech impairment (30%–60%), and epilepsy (20%) (33, 34). Consistently, in our cerebral infarction group, three patients had hemiparesis (75%) and one had epilepsy (25%). Two patients had language impairment (50%), both with right-sided brain lesions, which contrasted with previous studies, showing that left-sided brain lesions were more likely to cause language impairment (7). The discrepancies may be due to the early age of onset (16 days and 2 years) in our patients. Severe brain injury causes difficulties establishing language function rather than loss of function. A previous study has shown that cerebral infarction occurs in the middle cerebral artery region in more than 50% of the cases (35). In this cohort, three of the four children with cerebral infarction underwent MRA and all of them showed right supratentorial vascular lesions. Two had right middle cerebral artery infarction and one had right cerebral hemisphere multivessel infarction. Vascular lesions-related brain injuries are mostly distributed along the vessels (36). In the present group, 47% of the children with cerebral hemorrhage or infarction as the primary cause of CE had encephalomalacia located in the lateral hemisphere of the involved vessels, while the rest had bilateral hemispheric lesions. It was probably due to diffuse cerebral edema in the acute phase or combined brain injuries, such as HIE, infection, or prematurity.

Incontinentia pigmenti is a genetic disease caused by pathogenic mutations in the Xq11 and Xq28 chromosomes. The main manifestations are rashes or hyperpigmentation along the Blaschko line, and involvement of ectodermal tissues, such as teeth, eyes, hair, and nerves. Approximately 30% of the patients with incontinentia pigmenti present neurological symptoms, including developmental delay, intellectual disability, epilepsy, paralysis and ataxia, and MRI images show cerebral white matter lesions and cerebral hemorrhage (37). Consistently, in our study, two children with incontinentia pigmenti had postnatal rashes and recurrent seizures during the neonatal period. The MRI during follow-up showed extensive periventricular and subcortical white matter softening, ventricular dilatation deformation, and corpus callosum thinning.

**Figure 2:**
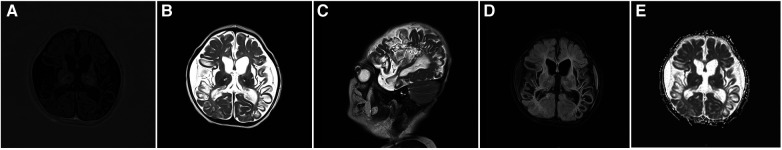
Cranial MRI images of a 3-month-old patient with hypoglycemia, hypoxic-ischemic encephalopathy, and cerebral hemorrhage. (**A**) Axial T1WI image shows reduced volume of bilateral temporo-occipital lobes, cortical contracture, and reduced signal. (**B,C**) Axial and sagittal T2WI images shows bilateral temporoparieto-occipital encephalomalacia and atrophy with partial cystic changes and bilateral temporoparietal subdural fluid accumulation. (**D**) Most high signals are suppressed at the cystic lesion site in the FLAIR sequence. (**E**) No significant restriction of water molecule diffusion at the cystic lesion site in the diffusion-weighted imaging sequence.

In the neonatal period, brain softening due to hypoglycemia is mostly observed in the parieto-occipital cortex and subcortical white matter areas of the cerebral hemispheres (38). In this cohort, the child with hypoglycemia (blood glucose = 2.1 mmol/L) also suffered from perinatal HIE and cerebral hemorrhage. Cranial MRI suggested bilateral cystic necrosis in the parieto-occipito-temporal region (Figure 2).

Our study has some limitations, such as a small sample size, the overlap of primary causes, and uncertain diagnosis of some cases. Secondly, treatment and rehabilitation may improve some clinical symptoms, such as paralysis and abnormal muscle tone, which then may affect the results. In the genetic disease group, we only have 2 children with pigment incontinence, which may not fully represent the characteristics of CE caused by various genetic conditions (e.g., methylmalonic acidemia, sulfite oxidase deficiency, propionic acidemia). Long-term prospective trials with large sample size are needed to further explore the clinical features of CE.

## Conclusion

When patients have HIE, intracranial infection, traumatic brain injury/hemorrhage, and ischemic stroke, the risk of developing CE should be considered, and close follow-up MRI is necessary. Delayed language or motor development, muscle tone disorders, seizures, and limb paralysis are common clinical manifestations of CE. Patients with HIE as the main primary disease have a higher proportion of patients with abnormal muscle tone disorders, while patients with ischemic stroke as the main primary disease have a greater proportion of patients with paralysis. There is no correlation between different primary diseases and the location of cystic lesions. These findings enrich the information about common etiologies and imaging characteristics of CE patients in the southwestern region of China.

## Data Availability

The raw data supporting the conclusions of this article will be made available by the authors, without undue reservation.
